# A National Department of Public Health in Relation to the Antituberculosis Problem1An address delivered before the International Congress on Tuberculosis, Washington, D. C., October 1, 1908.

**Published:** 1908-11

**Authors:** Charles A. L. Reed

**Affiliations:** Cincinnati, O.


### A National Department of Public Health in Relation to the Antituberculosis Problem.[1]

By CHARLES A. L. REED., M. D., Cincinnati, O.

(*Lancet-Clinic*, October 10, 1908.)

THE text of my address today is to be found in a few important facts. Tuberculosis alone causes the death of one hundred and forty thousand persons and the constant illness of two-thirds of a million more each year in the United States. The disease is preventable.

The United States today has no national public health agency vested with power competent to cope with the evil. Both of our great political parties have this year committed themselves to the creation of such a national public health agency, and yielding to

1. An address delivered before the International Congress on Tuberculosis, Washington, D. C., October 1, 1908.

the agitation inaugurated by Dr. C. G. Comegys, of Cincinnati, at the meeting of the American Medical Association in this city in 1891, and since continued by that organisation in connection with other organisations that have later joined in the movement, the President has signified his intention to bring to the attention of the Congress, at its session in December, specific proposals looking toward the correction of this unfortunate defect in our governmental organisation.

The object of my remarks is not to impress this great body of scientists from every quarter of the globe, for that is unnecessary, but through this body to impress the people of this country with the extreme importance of supporting these proposals, to the end that lives shall be saved, and that this shall be better done, that science shall be given due honor in the practical government of men.

### THE GREATEST HUMAN ASSET.

In approaching this question let us remember that the basis, the very fundamental basis, of prosperity is the productive energy of the people. We have been hearing much of our natural resources. The soil, the mines, the waterways and the forests have each been enumerated as an asset. Yet each of them would be as worthless as the sandy stretches of the Sahara without the developing power of the greatest of all our natural resources—human labor and human intelligence. It is, therefore, the human factor, first, last and all the time that counts. Yet I am sorry to say that it is a factor that is taken all too little into account by our publicists when they assume to talk learnedly on the great problems of national economics. This, I regret to say, arises too much from the fact that, with them as with the people generally, human life has become a mere emotion, and not much of that, and almost without being recognised as a unit of value translatable into terms of dollars and cents.

Yet I am confident that the chief national legislation of the immediate future, as already indicated, will be based upon the fact that a man's energy is his best asset, and that its full value depends upon his health and his length of years.

### SCATTERED NATIONAL HEALTH AGENCIES.

This movement is fostered not only by human and economic considerations, both of which I shall discuss at greater length later on, but by the fact, as already intimated, that the exising public health agencies established from time to time by our National Government, are insufficient and incoördinate and, to that extent and in consequence of that fact, are both unnecessarily expensive and disastrously inefficient in coping with this devastating evil.

In the first place, they are scattered throughout the various departments of the government. In consequence of this arrangement, or lack of arrangement, the Secretary of the Treasury is now the chief health officer of the United States. The Secretary of Commerce and Labor is in charge of the very fundamental basis of a health department,—namely, the bureau of vital statistics. The Secretary of Agriculture has the enforcement of the most important public health law that has recently been enacted, namely, that relating to pure foods and drugs, and likewise the bureau of animal industry. The Secretary of the Interior is the sanitary administrator of the territories, of the Indian tribes and of the great national eleemosynary institutions. The Secretary of War has charge of the sanitation of our insular possessions, including the Isthmian Canal Zone. The Secretary of the Navy and the Secretary of War, under the advice and direction of their respective surgeons general, are, of course, in control, as they ought to be, of the health of, respectively, the navy and the army. Many other instances might be given to illustrate the fact that our national public health agencies, considered in the aggregate, are in no possible sense connected with a coördinating center or under the direction of an intelligent and purposeful centralised administration.

### THINGS DONE AND NOT DONE.

This would be a matter of comparatively little concern if the results were not literally disastrous to the people. It is true that there are many things that are being done, and being well done, to protect the people against unnecessary disease and death. Our frontier quarantine is reasonably efficient; our protection against plague and pestilence of foreign origin seems satisfactory; our immigration inspection is good; our pure food and drug administration is eminently satisfactory, while the sanitary triumph in the Isthmian Canal Zone marks an epoch in the protection of human welfare.

There are, however, many things that are not done under existing conditions, things that ought to be done and would be done if we had a properly organised national department of public health.

### DISASTROUS WARFARE.

This is strikingly true of the great problem involved in the long, hard warfare here inaugurated in this country against an enemy, already encamped in our midst, more numerous than all the men in all the armies and all the navies of all the world in all the ages, an enemy that is triumphantly taking from the American people each year victims in death more than equivalent to one whole army and two whole navies of the United States. This enemy is the tubercle bacillus. But where one dies of tuberculosis, many are ill—how many it is quite impossible to tell in the absence of information that could be gathered only through the instrumentality of a great national health agency, such as is now under serious advisement in governmental circles. I have been led to believe that the ratio of mortality to morbidity in tuberculosis is as one to five. If this is even approximately true, it follows that each year while 140,000 people are dying of this disease 700,000 are ill from the same cause. Invalidism induced by this cause is generally prolonged, varying from a few months to several years. One year is under the mean average duration of the disease. What does this mean? Statistics relating to a general condition spread all over three millions of square miles are not so impressive as if the same condition were concentrated and restricted to a more limited area.

### SOME STRIKING ILLUSTRATIONS.

Let us say, then, that every man, woman and child in the State of Connecticut is stricken with this disease and remains an invalid for the space of an entire year, and that the deaths among them represented the entire population of New Haven with half the population of Hartford. Or let us imagine that Indianapolis or St. Paul or Kansas City had suddenly dropped off the map. The disparity in population between these cities and the deaths that occur annually from tuberculosis is not sufficient to destroy the force of the comparison for the purpose of an object lesson.

### WHAT IS IT WORTH?

This sounds like strong language, it doubtless is strong language, but I challenge refutation of its accuracy. But strong as are the statements, startling as must be the illustrations, they will, I fear, fail to arouse action unless their meaning is expressed in dollars; it is not worth while to bother about the cents. In this computation our unit must be a human life. What is it worth? I am not asking the widow or the orphan. I am asking our publicists; I am asking the hundreds—yes, literally hundreds of lawyers in the Congress. But I ask them in vain. Their published utterances relate to money—always money—tariff money, railroad money, labor money, Standard Oil money, to all kinds of money except the money that is represented in the very life of the money-earner himself. On this they are silent. So we must begin for ourselves.

### THE EQUIVALENT IN COIN.

A man's life is his capital; what he earns is the interest on his capital. Most states make 6 per cent. a legal rate of interest.

But make it 5, both for conservatism and easy counting. Then compute a man's wage, say a dollar and a quarter a day for 300 days in the year, as 5 per cent. on his capital. Then estimate on that basis the value of 140,000 lives lost—capital and interest—and the value of the time of 700,000 persons for one year, and you begin to grasp the colossal money phase of this question viewed in its strictly economic aspects. Of course, many who die from tuberculosis are dependents, many are non-producers, and many who are producers do not earn a dollar and a quarter a day, and many do not work 300 days a year. But it is likewise true that many, very many, of these victims have an earning capacity running high up into the thousands of dollars—from a single thousand up to half a million.

So it is probable that our units of computation are not far out of the way. But still to be conservative, divide the aggregate loss thus arrived at by two and again by two and you will still have enough left, which, if turned into the national treasury, would defray the running expenses of army and navy, build our coast defenses, improve our waterways, build the Panama Canal and pay the national debt long before our outstanding obligations mature.

### STATE AND FEDERAL CONTROL.

In considering the question of a national department of public health in its relation to the control of tuberculosis one of the first conditions to be considered is the limitation, under the form of government, of the state and national governments, respectively, in the premises. It may be said that practically everything that is being done in the tuberculosis problem today is being done by the states by virtue of the police power reserved to them under the constitution. This fact, more than any other one aside from alcohol and popular apathy, is probably responsible for the comparative dearth of results so far achieved. In the first place the status accorded to sanitary authority in the states, as it is in the National Government, is very generally, but not always such as to give it neither educational value, moral force or adequate executive authority.

### UNIFORM LAWS—A COUNCIL OF STATES.

The laws in the different states are generally inadequate, and, whether adequate or not, their complete lack of uniformity renders it impossible for the states to coöperate in the solution of a problem which, like this problem, is co-extensive with all the states. Like laws for like conditions is the first requisite for the control of any national necessity by state legislation. And like laws by the states will probably not be enacted to any great extent until, under the initiative of the National Government, the states shall send delegates to a representative body, a sort of council of states, which shall meet, as a legislature or the congress meets, in a session or sessions long enough to accomplish satisfactory results, and whose object shall be to formulate standard bills on various subjects and send them back to the legislatures for enactment. In this way, and in this way alone, can the states move with anything like satisfactory rapidity in meeting the crying demand for like laws for like conditions not only as relates to great sanitary problems, including the problem of tuberculosis, but relative to many other problems that concern the economic and social welfare of the people. This step should be taken without delay for, even at the best, considerable time must elapse before results from this means can be realised.

In the absence of some such effort on the part of the states, or even with such effort, but recognising that there is enough for both state and federal government to do, it seems to me that the congress, acting under the general welfare clause of the constitution, is competent to take charge of the situation. This principle was recognised by the conference of governors of the southern states, held only a few years ago, and has been adopted and acted upon by the general government as applied not only to maritime but to inland and inter-state quarantine of yellow fever. Acting on this principle and along lines parallel with those followed in the instances of yellow fever and the bubonic plague, it becomes entirely competent for the National Government, when necessary, to take charge of domiciliatory sanitation, the absence of which is the one factor most responsible for the propagation of tuberculosis. The National Government has the only power competent to enforce sanitation of railway coaches and steamboats engaged in inter-state business, and of steamships touching our ports. The Pullman coaches, next to tenement houses, it would seem from a study of all conditions, are, probably, the most potent factors in the dissemination of tuberculosis. The federal government should have as much control over the migration of tuberculosis patients as over any other disseminators of a plague. The question of establishing great sanatoria on government land reserves, and of colonising them with tuberculosis patients from the crowded centers is another detail, but one that can be solved best probably by the coöperation of state and national authorities. And this reminds me to say that in all this great campaign the dominant idea must be that of coöperation rather than antagonism between state and federal authority, and that in every instance in which conflicting laws prevent such a consummation a way must be found by coöperative action to change the laws. This is not the land of the Medes, neither of the Persians.

### A MATTER OF PRACTICAL LEGISLATION.

Then by all means let us have this dual plan of campaign against tuberculosis. Let us attempt, through a council of states, to secure uniform state laws touching this and other important questions. And let us have a strong federal public health agency with which to do the national side of the work. About this latter proposition it seems to me there ought to be no particular difficulty. The existing national public health agencies could be assembled by transfer from the various departments in which they are now located into some one of the existing departments and there organised, under authority of additional law on the basis of highest efficiency.

### THE PARAMOUNT QUESTION OF STATUS.

I have said that these agencies should be assembled in some existing department rather than organised into a separate and distinct department with representation in the cabinet. I have said this because there appear to be sound executive reasons why the cabinet should not be enlarged. At the same time I must insist that the fundamental importance of this health movement, as shown by this congress, and by hundreds of other congresses and associations, demands that, for its fullest efficiency, it should be accorded the educational and moral force to be derived only from its titular recognition in the departmental scheme of our government. The two propositions are fortunately readily reconciled. This can be done by changing the name of the department in which the health agencies are assembled, thus avoiding the multiplication of cabinet officers. Thus, if it shall be found wise to make the bureau of vital statistics the nucleus for the aggregated service, the name of the department in which it is located could be changed to that of "commerce, labor and public health." Or if it shall be deeemed expedient to select the department of the interior the name of that department should be changed to that of education and public health, which, unlike the present title would mean and stand for something distinctive among our great national interests. But I insist, in the name of the medical profession, in the name of science, in the name of humanity, that a great governmental agency to conserve, as this one proposes to conserve, more lives, more homes, more property, and more money, than any other single agency in the whole plan of government, shall be accorded a status befitting its dignity and its worth to all the people.

The Groton.

